# Painting image browser applying an associate-rule-aware multidimensional data visualization technique

**DOI:** 10.1186/s42492-019-0040-7

**Published:** 2020-02-05

**Authors:** Ayaka Kaneko, Akiko Komatsu, Takayuki Itoh, Florence Ying Wang

**Affiliations:** 1 Ochanomizu University, 2-1-1 Otsuka, Bunkyo-ku, Tokyo, 1128610 Japan; 2 CSIRO, Julius Ave., North Ryde,0 NSW, 2113 Australia

**Keywords:** Painting image, Multi-dimensional data visualization, Association rule

## Abstract

Exploration of artworks is enjoyable but often time consuming. For example, it is not always easy to discover the favorite types of unknown painting works. It is not also always easy to explore unpopular painting works which looks similar to painting works created by famous artists. This paper presents a painting image browser which assists the explorative discovery of user-interested painting works. The presented browser applies a new multidimensional data visualization technique that highlights particular ranges of particular numeric values based on association rules to suggest cues to find favorite painting images. This study assumes a large number of painting images are provided where categorical information (e.g., names of artists, created year) is assigned to the images. The presented system firstly calculates the feature values of the images as a preprocessing step. Then the browser visualizes the multidimensional feature values as a heatmap and highlights association rules discovered from the relationships between the feature values and categorical information. This mechanism enables users to explore favorite painting images or painting images that look similar to famous painting works. Our case study and user evaluation demonstrates the effectiveness of the presented image browser.

## Introduction

The recent evolution of image search technologies realized easy search of painting works. We can search for our favorite painting works by queries with keywords or similar images. This mechanism is effective if we know appropriate words or we have similar images. In other words, other types of mechanisms are desirable when we do not know appropriate query words and also we do not have images similar to our demanded painting works. We may sometimes want to search for our favorite painting works which we do not know the name of artists or created years. Or, we may sometimes want to discover painting works that similarly look like famous painting works. In such situations, interactive and exploratory painting image browsers would be effective to realize flexible and enjoyable processes of painting image search.

Content-based image retrieval (CBIR) techniques have utilized both categorical information such as metadata and annotations of images and numeric information such as feature vectors calculated from pixel values of the images. Image features have been well studied with various visual properties including colors, shapes, and textures. Nowadays, we do not need to design such hand-craft feature spaces while applying deep learning techniques to retrieve user-interested images. But still, hand-craft features are useful when we cannot prepare large training datasets or require intuitive understanding and explanations. Visualization of such feature values is an effective approach to navigate the painting image collections since hand-craft features are understandable and explainable.

Multidimensional data visualization is important for various fields of science and business, and therefore a lot of visualization techniques have been presented. Multidimensional datasets in our daily life often contain numeric, ordinal, and categorical values [1]. For example, datasets of professional baseball players may contain various performance values described as numeric values, and attributes such as positions or names of teams as categorical values. Many studies have applied association rule mining techniques [2] to discover essential relationships among such variables. Multidimensional data visualization techniques are expected to assist in discovering and understanding the relationships among the variables while working with association rule mining techniques.

We present a visualization technique which assists users to visually find association rules from multidimensional datasets. This technique draws a set of parallel axes mapped to numeric variables. The technique also divides the axes into a predefined number of intervals and paints small component bar charts there. It brightly paints the component bar charts if their corresponding ranges of the numeric variables satisfy association rules.

We have also extended the visualization technique as a painting image browser. This application visualizes a painting image dataset which contains images themselves and their multidimensional values. Image features are treated as numeric values while textual attributes of the images are associated as categorical values in this browser. The browser highlights particular ranges of image features if they satisfy association rules. The browser also provides interaction mechanisms to assist users to discover preferable painting images looking at the highlighted association rules.

### Association rule and visualization

Several visualization techniques [3, 4] have been presented to realize the representation of association rules. On the other hand, there have been few studies that simultaneously represents the distribution of numeric values as well as confidence and support values of association rules.

The study of visualization technique presented in this paper is inspired by our previous multidimensional data visualization technique [5] which displays multiple low-dimensional spaces as small Parallel Coordinate Plots. The technique applies two types of dimension set selection methods. One of the methods extracts sets of dimensions that have large positive/negative correlations. The other extracts sets of dimensions that satisfy particular association rules with a user-specified categorical variable.

Our previous technique is one of the visualization techniques which represented association rules aggressively. But still, the technique did not clearly represent which ranges of numeric variables satisfy the association rules. We addressed this issue and developed a new visualization technique that highlights the particular ranges of numeric variables that satisfy the association rules.

### Image browser and visualization

We extended the presented visualization technique as a painting image browser based on the assumption that the features and annotations of painting images are helpful information to assist users to discover their favorite painting images. In other words, the image browser treats image features as numeric variables while image annotations as categorical variables and then emphatically represents the association rules.

There have been a large number of studies on image browsers. Bederson [6] and Gomi et al. [7] presented typical studies that display hierarchically structured images. These techniques configure the layout of hierarchically structured images by applying space-filling algorithms.

Several techniques for browsing images as unstructured datasets have been also presented. Semantic image browser [8] applies a dimension reduction scheme while D-FLIP [9] applies a force-directed algorithm.

ImageCube [10] supposes that images have multi-dimensional vectors. ImageCube assigns two variables onto horizontal and vertical axes to place a set of images. Users can discover the relationships between the appearances of images and the multidimensional values associated with the images.

### Painting image processing

Hand-craft image features for painting works have been well-discussed [11, 12]. Most of the studies have applied combination of various visual properties including color, edge, texture, composition, and feature points to calculate the hand-craft feature values. Such features have been applied to various image processing tasks such as classification and clustering as recent deep learning technologies have been also applied

There have been several studies on browsing software for painting images [13]. In our survey, we did not find any studies focusing on association rules between categorical and numeric information assigned to painting images.

## Methods

We present a painting image browser in this paper. This browser applies a multidimensional data visualization technique that navigates into user-interested portions of the input datasets based on association rules. The visualization technique presented in this paper highlights the ranges of numeric variables if they satisfy association rules.

In this section, we firstly describe the data structure and processing flow of the visualization technique. Then, we present the painting image browser as an application of the presented visualization technique. This browser treats annotations as categorical variables while image features as numeric and variables. The browser assists users to explore sets of images while applying association rules as clues of image exploration.

### Data structure and process flow

This paper defines that a multidimensional dataset *D* contains *n* individuals. Also, the paper supposes individual *a*_*i*_ has *m*_*v*_ real variables and *m*_*c*_ categorical variables. We describe the data structure as follows:

1 $$\begin{array}{@{}rcl@{}} D= \{a_{1}, a_{2},..., a_{n}\}  \\ a_{i}= \{v_{i1}, v_{i2},..., v_{im_{v}}, c_{i1}, c_{i2},..., c_{im_{c}} \} \end{array} $$

where *v*_*ij*_ is the *j*th real variable of the *i*th individual, and *c*_*ik*_ is the *k*th categorical variable of the *i*th individual.

**Fig. 1:**
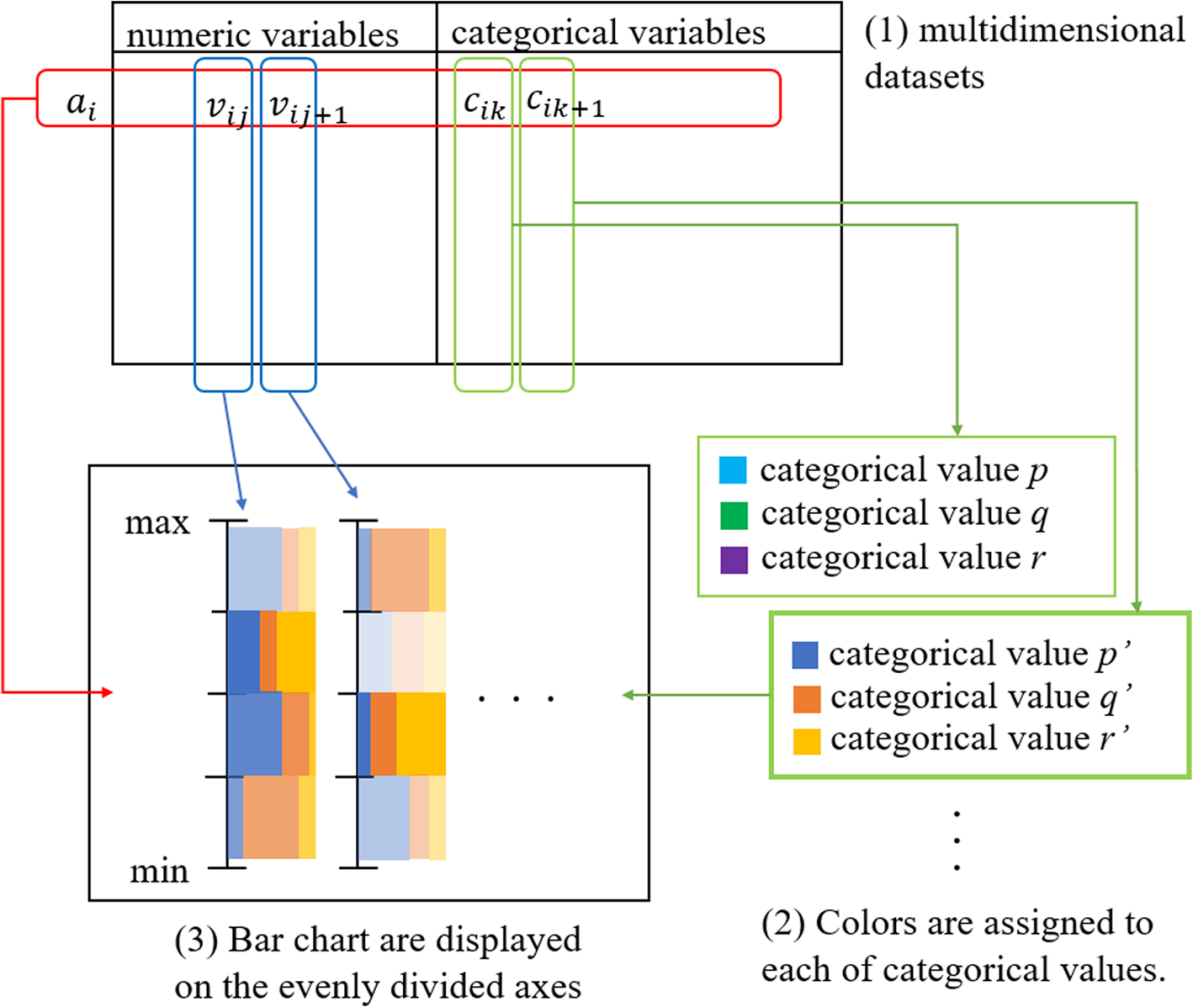
Processing flow of the presented technique. **1** multidimensional datasets. **2** Colors are assigned to each of categorical values. **3** Bar chart are displayed on the evenly divided axes

The processing flow of the presented technique is illustrated in Fig. 1. Figure 1(2) depicts that the technique assigns colors to categorical values of the user-specified variable. It draws a set of axes corresponding to real variables, divides the axes into *k* intervals, and paints small component bar charts at each range, as shown in Fig. 1(3). The ratios of categorical values are depicted by the component bar charts. The intensity of the component bar charts represents the number of individuals in the ranges corresponding to the component bar chart. Users can understand the support and confidence values of the association rules by observing this representation of component bar charts.

### Association rule mining

This technique finds the combinations of the categorical values and the ranges of numeric variables that satisfy association rules. Here, let *A* be a particular range of a numeric variable, and *B* be a particular categorical value. This paper describes an association rule by *A* and *B* as follows:

*A*→*B* or *B*→*A*

The above definition indicates that many individuals have the categorical value *B* in the range *A*.

The technique calculates the support *P*_*sup*_ and the confidence *P*_*con*_ for an arbitrary pair of *A* and *B* by the following equations:

2 $$\begin{array}{@{}rcl@{}} P_{sup}=P(A,B)  \\ P_{con}=(B|A) \end{array} $$

*A* and *B* are extracted if both *P*_*sup*_ and *P*_*con*_ are larger than the user-specified thresholds.

The component bar charts corresponding to the ranges that satisfy the above conditions contain large portions of specific categorical values and are painted brightly. It means that our representation of component bar charts depicts how the ranges of numeric variables satisfy association rules.

Thresholds of support and confidence values are interactively adjustable using slider bars in our implementation. In other words, users can adjust the number of ranges satisfying the association rules using the slider bars.

### Hand-crafted image features

Due to the evolution of CBIR techniques, image feature design has also been an active research topic. Zhou et al. [14] introduced algorithms and image features for CBIR. Image features include global features based on color, shape, texture, and composition, as well as local features such as scale-invariant feature transform [15]. Such conventional image features are often termed "hand-crafted features". On the contrary, abstract representations of image features constructed by deep neural networks have been recently studied and applied to image retrieval techniques.

We implemented hand-crafted features in this study because we prefer explainable feature values for painting image exploration.

The list of hand-craft features applied in this study is shown in Table 1 (reproduced from ref. [16]). We applied the global features such as colors, edges, and compositions which Wang [17] proposed, in addition to the local image features based on DoG filter, Gabor filter, and local color histogram [18]. We implemented the color temperature score and color weight score as color features since Wang [17] had good results with these features. Color temperature of indicates the feel of warmth or coldness of colors. Color weight indicating the feel of heaviness of color is another criterion in assessing colors. We subjectively supposed these features effective for painting image search. Further, images are decomposed into a pre-defined number of local colors before calculating the temperature and weight for each local color. The number of local colors was three in our study, since we are inspired by ref. [19], in which statistics of three largest segments of a painting are used as features for evaluating its aesthetic quality. In addition, we calculate the line feature from the number of edges extracted by the Hough function. Composition feature is calculated from the saliency model presented by Itti and Koch [20].

**Table 1:** Image feature applied in this study

Feature value	Description	Dimensions
Color temperature	Visual temperature of	3
	color of three segments	
Color weight	Visual weight of color	3
	of three segments	
Line	Straight line ratio,	4
	mean slope, mean length,	
	standard deviation of slopes	
Composition	Mean saliency for each	9
	of the nine image regions	
	divided by "Rule of thirds"	
Gabor filter	Multi-directional high	40
	frequency component	
DoG filter	Differential feature values	6
	independent of direction	
Local histogram	Local gray-scale histogram	90

Some image features such as color temperature score, color weight score, and line associate the global appearance and impression of painting images. Thus, we treated these features as numeric values while visualizing them. Meanwhile, we apply a clustering algorithm to divide images based on the composition feature. Also, we implemented some local features including Gabor filter, DoG filter, and local histogram since Kuriyama [18] had good results with these features. These local features are also used for the clustering process. Then, the cluster IDs of images are used as categorical variables in our browser. The browser applies a principal component analysis with each of the above image features in order and then generates clusters by the k-means algorithm. The Davies-Bouldin Index [21] is applied to measure the quality of the clustering results and finally specify the optimal number of clusters.

### User interface components

**Fig. 2:**
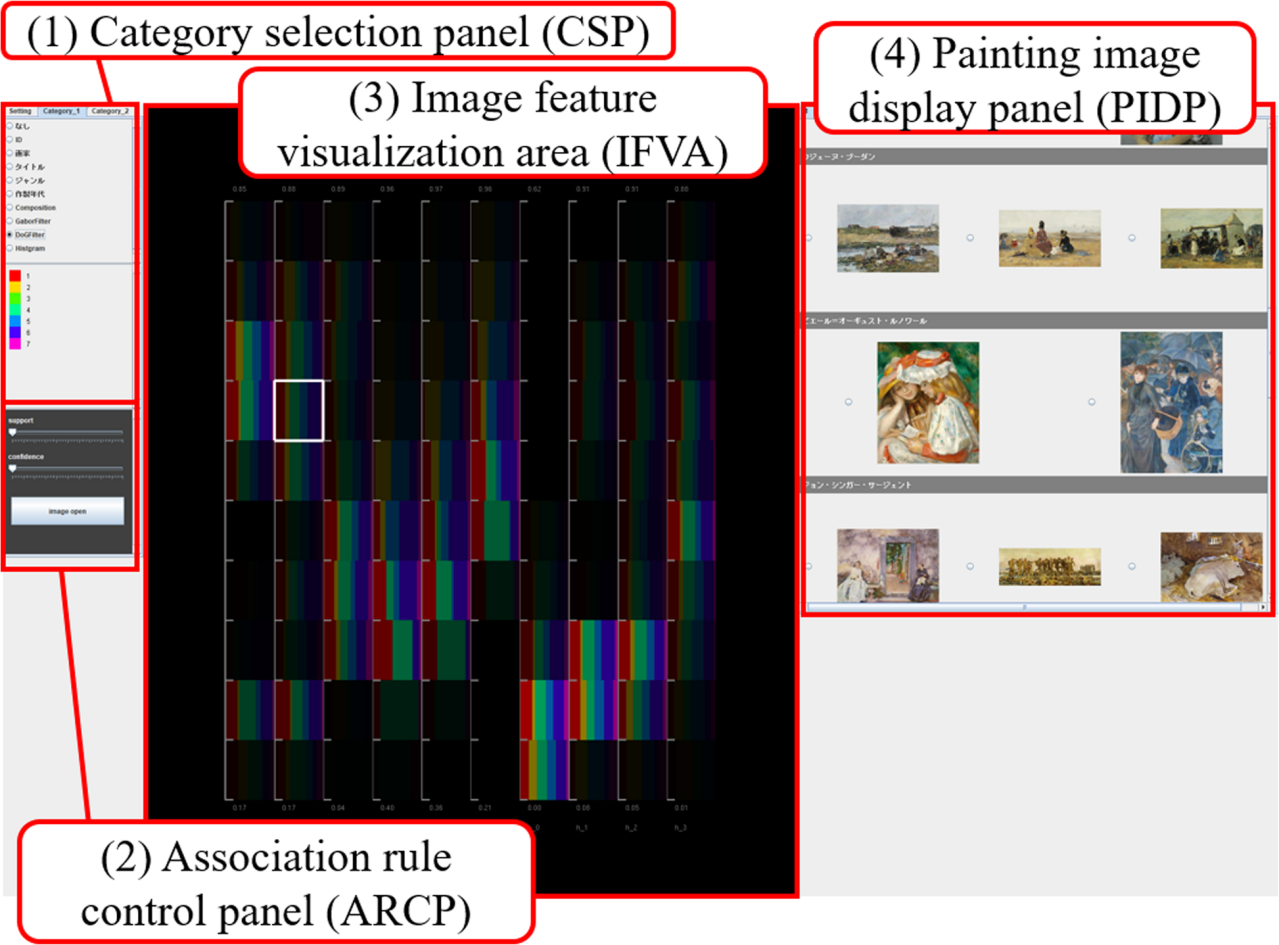
Snapshot of our application consisting of four user interface components. **1** Category selection panel (CSP). **2** Association rule control panel (ARCP). **3** Image feature visualization area (IFVA). **4** Painting image display panel (PIDP)

The user interface of our painting image browser is shown in Fig. 2. We developed the following four components on the user interface:

- Category selection panel (CSP) (Fig. 2(1))
- Association rule control panel (ARCP) (Fig. 2(2))
- Image feature visualization area (IFVA) (Fig. 2(3))
- Painting image display panel (PIDP) (Fig. 2(4))

**Fig. 3:**
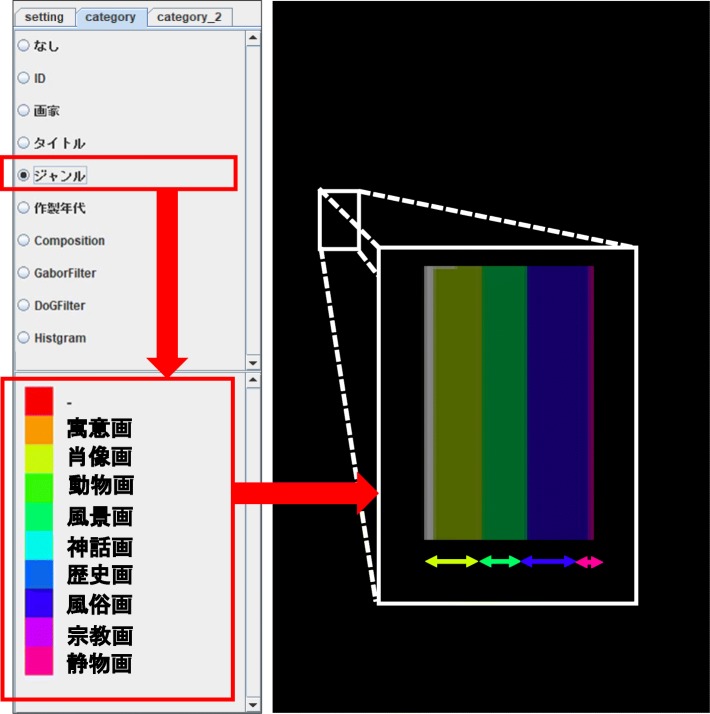
Categorical variable selection by radio buttons. The color palette displays a list of colors and corresponding categorical values. Component bar charts visualize image feature values

The IFVA draws component bar charts to visualize the image features as numeric variables. The component bar charts represent the distribution of a categorical variable interactively selected on the CSP as shown in Fig. 3. Radio buttons are featured at the upper part of the panel so that users can interactively select categorical variables. The panel also indicates categorical values and their corresponding colors at the lower part; subsequently, the browser updates the component bar charts in the IFVA.

The brightness values of the component bar charts are calculated from the total number of images contained in the corresponding ranges. The widths of the colored subregions in the component bar charts are calculated from the percentage of images belonging to a particular category corresponding to a particular color. Users can easily find major categories of major ranges since the corresponding subregions are brightly and widely painted. Here, the confidence values of the association rules correspond to the widths of the subregions, whereas the support values correspond to the brightness values.

**Fig. 4:**
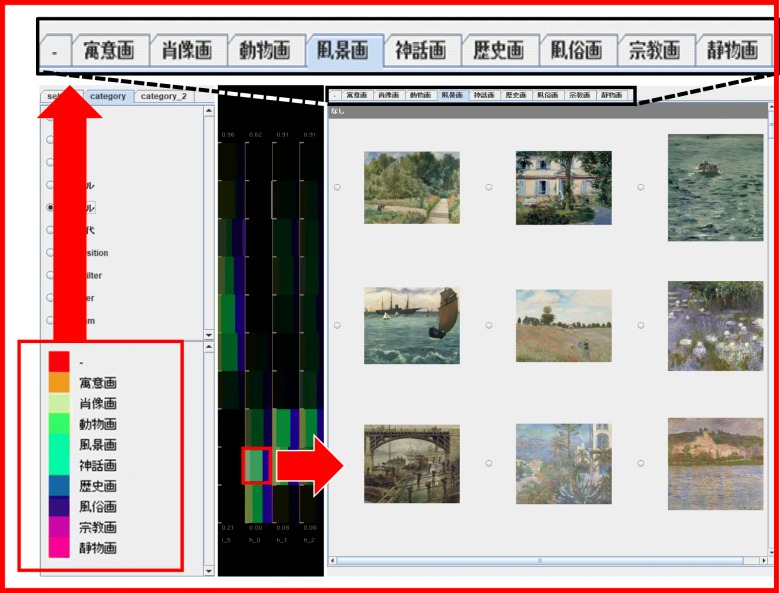
Corresponding painting images are displayed according to the click operations of component bar charts

**Fig. 5:**
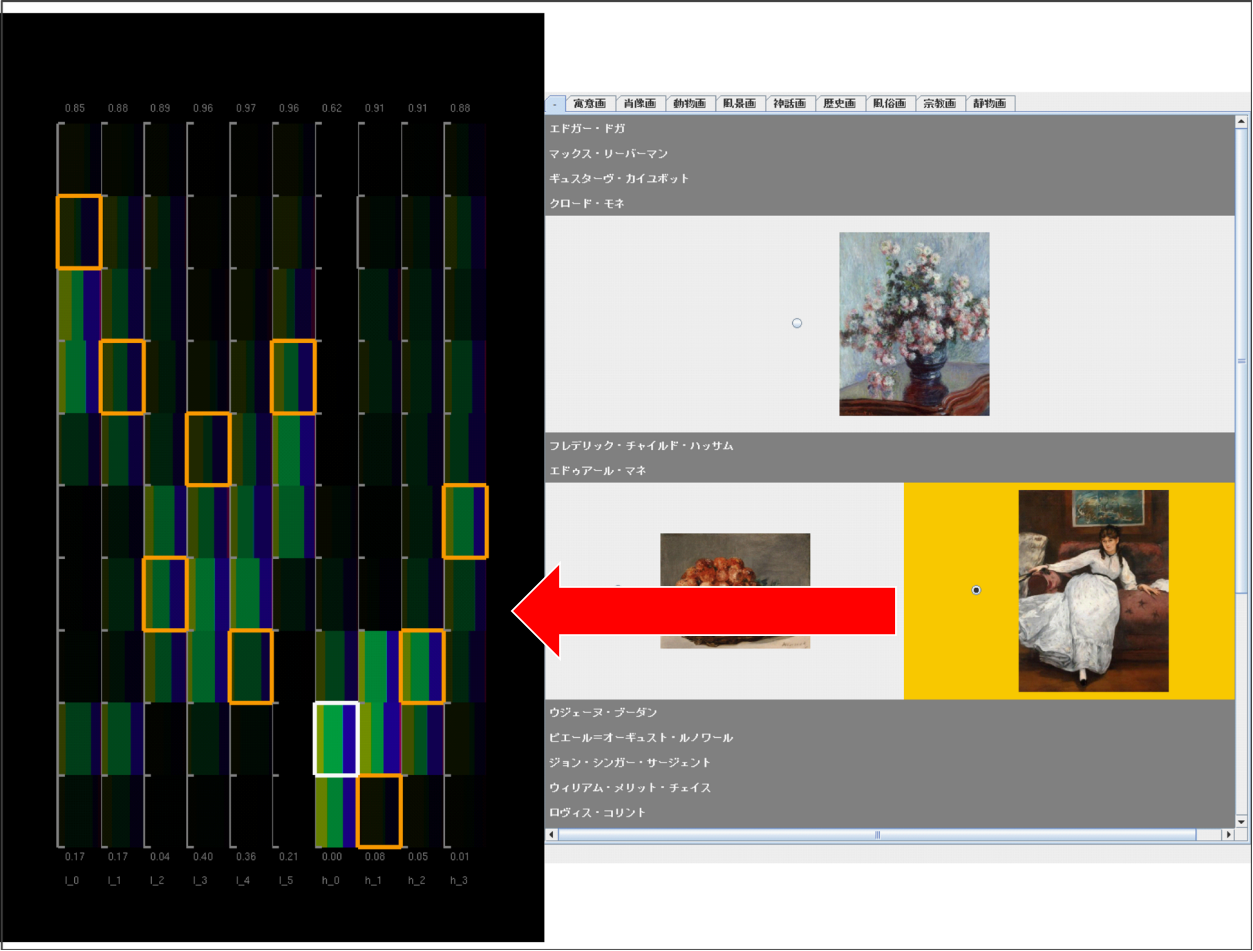
Subranges corresponding to the user-selected painting image are highlighted by orange borderlines

Figure 4 shows that this browser displays a set of painting images on the PIDP when a user clicks on a particular component bar chart. This panel generates multiple tabs to display the painting images. The categorical values of the user-specified categorical variable are associated with the tabs. Painting images are divided based on their categorical values and then displayed on each of the corresponding tabs. Here, the browser sorts the displayed images based on another categorical variable selected by the users on the CSP. The former categorical variable is called “coloring category” while the latter variable is called “sorting category” in this section. Figure 5 shows that the IFVA marks the component bar charts corresponding to the user-selected painting image; these highlights perform as clues for users to find similar images.

**Fig. 6:**
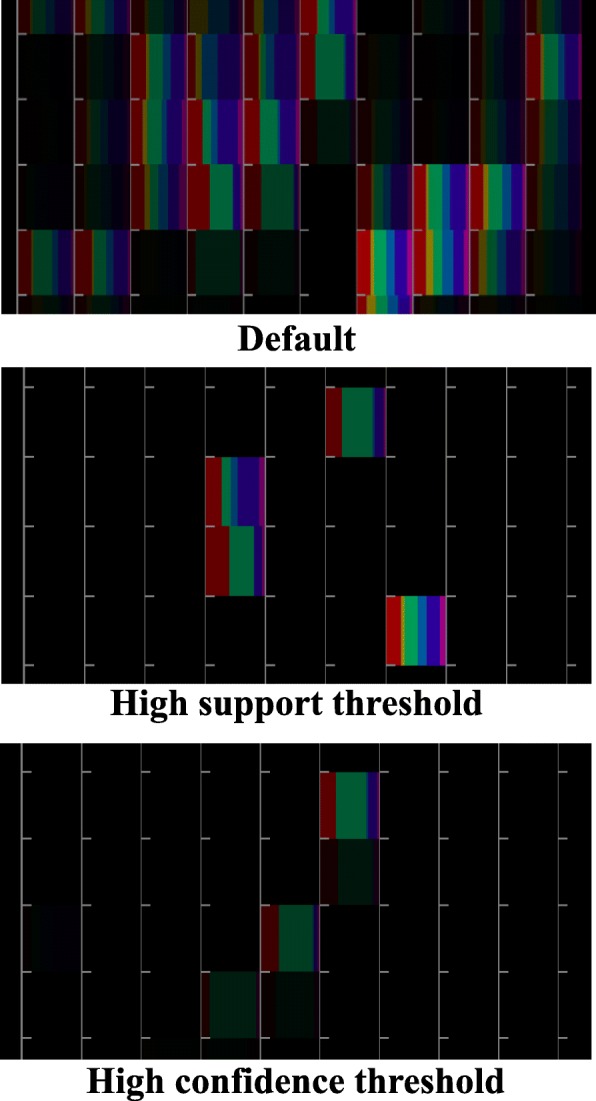
Threshold adjustment of association rule by using slider bars

We provide slider bars to control the thresholds of confidence and support values on the ARCP. Also, the component bar charts corresponding to the ranges that do not satisfy any association rules can be deleted. Figure 6 shows the operation of thresholds. The browser highlights the ranges containing many images when a user sets the threshold of the support value larger. Furthermore, it highlights the ranges containing the specific category of many images when the user sets the threshold of confidence value larger.

## Results and discussion

### Image collection

We constructed an image collection dataset containing the name of image files, image feature values, clustering results of some image feature values, and annotations including artist name, year of production, and genre of the painting. The dataset contains 858 painting images downloaded from a public domain website [22], which are annotated as “impressionism.”

The variables and annotations contained in the dataset are listed as follows, where the details of the image features are explained in Table 1:

- Categorical variables
  - Annotations (artist name, year of production, genre of the painting)
  - Clusters (Gabor filter, DoG filter, local histogram, composition)
- Real variables
  - Color temperature score, color weight score, line score

After measuring the quality of clustering results, we selected the number of clusters with composition as seven, with Gabor filter as six, with DoG filter as seven, and with the local histogram as two.

This dataset is constructed based on our target that we would like to navigate users into the image collections and assist them to discover their favorite images with the following conditions:

- Discover their favorite painting images, whose names and artists are unknown.
- Discover unknown paintings that look similar to favorite famous paintings of the users.

Annotations can be good references while image features can act as clues to find their favorite paintings. The presented browser visualizes them to assist users in understanding the properties of their favorite paintings, and reuse this knowledge as clues while exploring the image collections.

### Case study

This section introduces a case study with the presented browser and discusses the corresponding actual scenarios. Here, this case study supposes that a user wants to discover preferable painting images with no query words or query images. The image dataset introduced in the previous section was used in this case study.

**Fig. 7:**
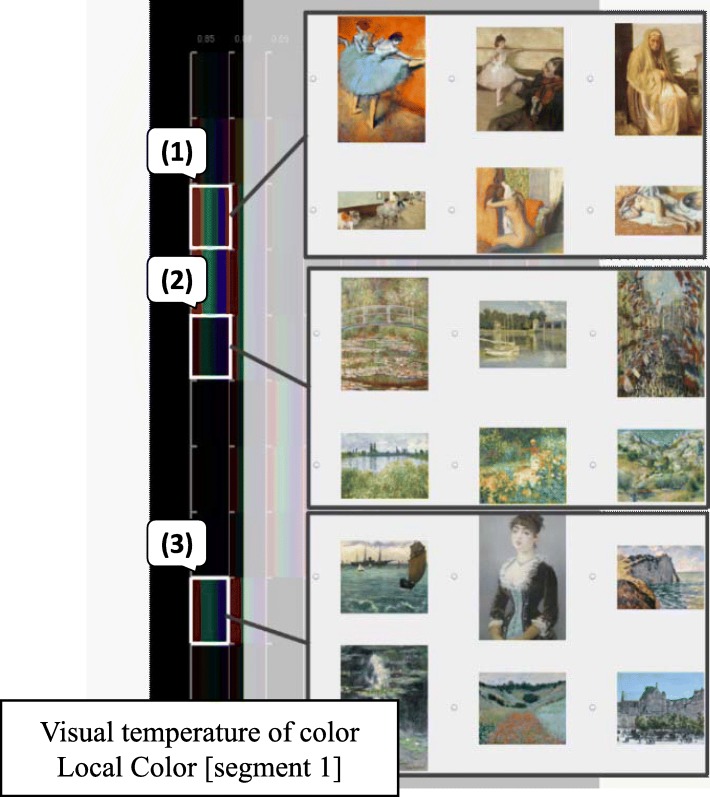
Visualization of color temperature score of the first local colors. Reddish, greenish, and blueish colors correspond to (**1**), (**2**), and (**3**) in this image

The user observed the IFVA first of all. The axis on the left end of the IFVA visualizes the color temperature score of the first local color. The images are divided into three types as shown in Fig. 7. Many reddish images belonged to the range (1) while the range (2) contained greenish painting images. The blueish images that the user preferred were in the range (3).

**Fig. 8:**
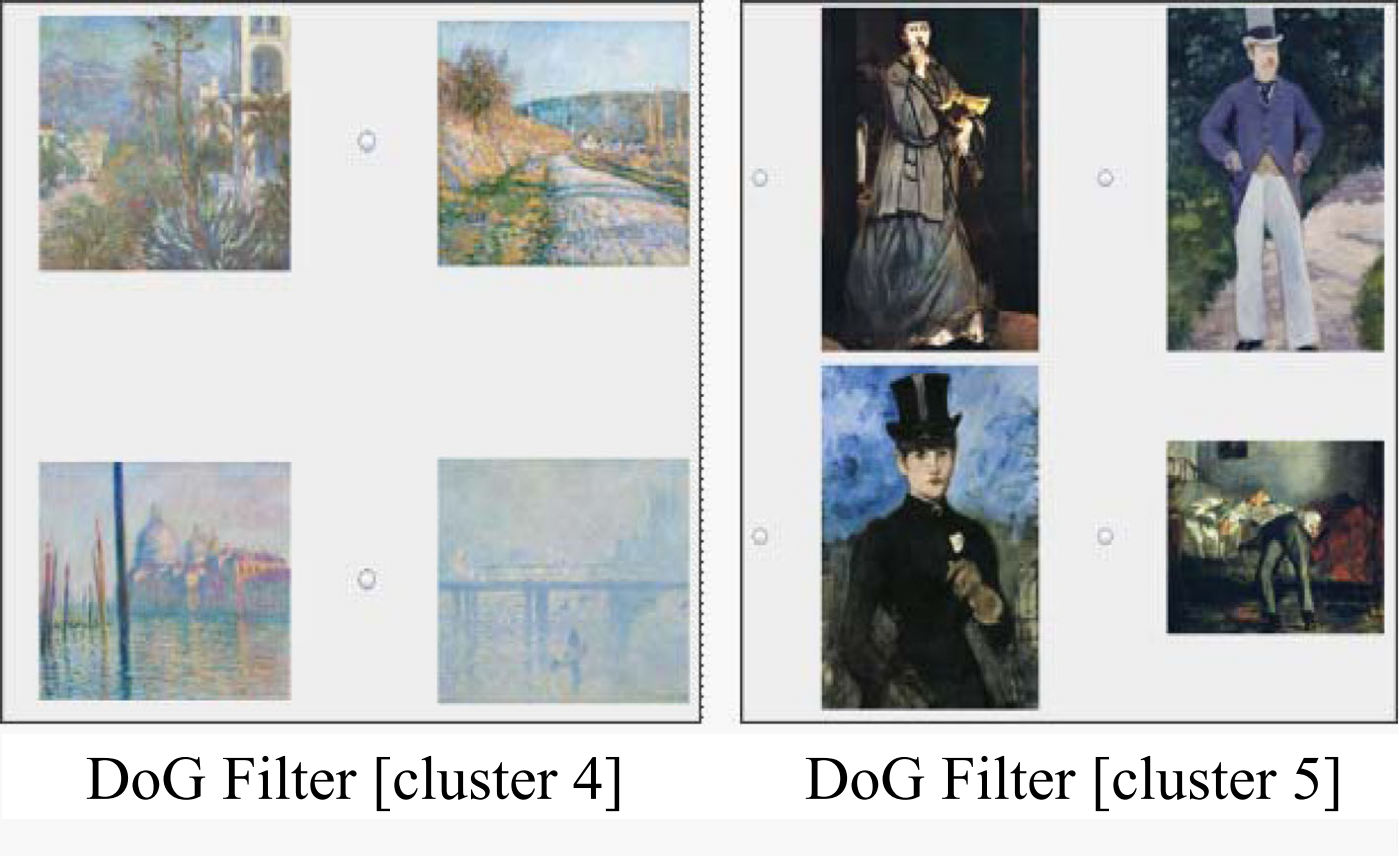
Painting images clustered based on the DoG filter feature values

The user observed the image features of the blueish images selecting the DoG filter as the coloring category. The browser painted the component bar charts in seven colors that correspond to the seven clusters divided based on the feature values of the DoG filter. The PIDP generated seven tabs associated with the seven clusters and displayed the images in each of the clusters. Example images belonging to clusters 4 and 5 are shown in Fig. 8. The cluster 4 contained many images that have a pale blue background and soft edges. Meanwhile, the cluster 5 contained many images that have vivid colors and clear edges. Alternatively, we could appreciate the painting images based on the user-selected categories as the sorting category on the CSP.

**Fig. 9:**
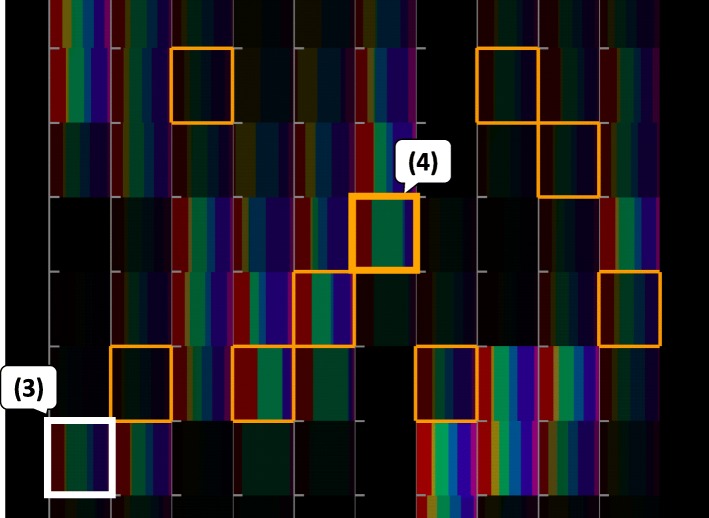
Visual discovery of association rules based on the distribution and the intensity of component bar charts

The user then selected a preferred painting image and searched for others that have similar features. Figure 9 shows that orange borderlines are drawn around the component bar charts corresponding to the ranges of numeric variables to which the selected image belongs. Figure 9 shows the range (4) that contains many images belonging to the cluster 4 corresponding to the large green part in the component bar chart. Also, this range contains a large number of images because the corresponding component bar chart is rendered brightly. This suggests that the cluster 4 satisfies an association rule with this range. Here, the axis of the range (4) represents the color weight score of the third color; this indicates that the range (4) contains images painted in light colors. This result demonstrates that the appearance of IFVA acts as clues to users in discovering the painting images that look similar to their favorite images.

This browser enables users to explore the set of desired images with their feature values and category information. Thus, users can find their preferable images by comparing the images that have similar features or belong to the same category. Also, the association rules are effective as clues to search for images that are similar to user-interested images.

### Experimental method

We conducted a user experiment with 14 female participants, who are graduate students at a women’s university, majoring in computer science. We explained the following task to all the participants: select their own best painting work from the image collection for displaying in a particular room known to all the participants. Further, we provided them with two image browsing software: the proposed browser and ImageCube [10], and instructed the participants to select their own best image. We recorded the final user-selected categorical values and the ranges of numeric values, when the users selected their own best images. Following this task, we directed the participants to evaluate the two browsers based on the five-points Likert scale from the perspectives of “browsing overview,” “narrowing down,” and “comparative browsing.”

We categorized the 858 painting images into X and Y collections randomly, and divided the participants into the following four groups: groups A and B had four participants, whereas the other groups had three participants each. Group A: ImageCube with collection X first, followed by the presented browser with collection Y. Group B: ImageCube with collection Y first, followed by the presented browser with collection X. Group C: Presented browser with collection X first, followed by ImageCube with collection Y. Group D: Presented browser with collection Y first, followed by ImageCube with collection X.

### Results and discussion of user evaluation

**Fig. 10:**
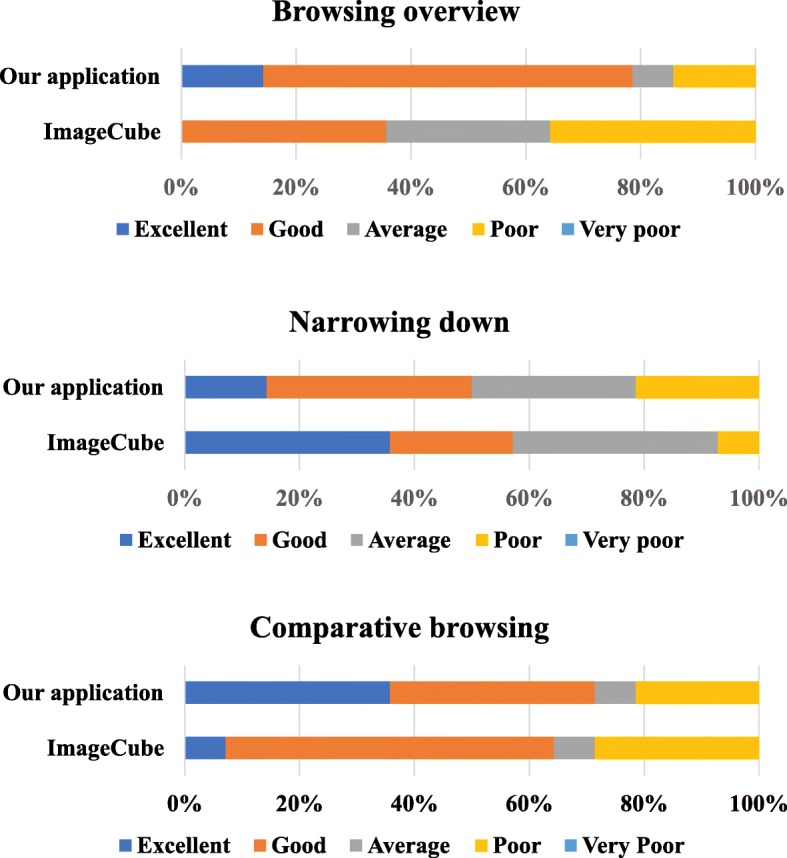
User evaluation results of the application for painting image collections

Figure 10 shows the statistics of user evaluation on “browsing overview,” “narrowing down,” and “comparative browsing.” It indicates that the presented browser received a higher evaluation for “browsing overview” and “comparative browsing.” We received several comments that scatterplot-based image browsers, including ImageCube, often caused visual cluttering because the images are overlapped, making it difficult to overview the image collections. In addition, we received several positive comments, such as “it is helpful in comparing the image features of particular images with others” and “it is easy to understand the number of images in each category,” regarding the color representation of the IFVA. We suppose that this visual representation of the relationship between categorical and numeric values made it easier for users to compare the images in detail. Further, ImageCube received several positive comments such as “scatterplot-based representation of ImageCube makes it easy for us to understand the distribution of numeric values with images.” We suppose that this feature of ImageCube is useful to narrow down the ranges of numeric values by zooming in on the user-interested regions of the visualization space.

We received several positive comments on association rule representation, such as “it is easy to find particular ranges of numeric values that have an unbalanced distribution of categorical values,” and “it is easy to focus on brightly painted ranges of numeric values that have higher support.” These comments suggest that the representation of confidence and support of association rules of our browser is useful to specify the user-interested regions in the image collections.

However, this evaluation does not ensure the effectiveness of the association rules in assisting the narrowing down operations of image collections. We observed that the association rules were helpful for several participants, whereas they did not work well for others. For example, a participant selected a range of numeric values that had higher brightness and an unbalanced distribution of categorical values, when she selected her own best image. She mentioned that this range was easy to find, and thus expected to find her interested painting works in this range. This result suggests that this participant carefully observed confidence and support values as the criteria to select her own best image. Meanwhile, the ranges selected by some of the other participants did not have higher confidence or support values, suggesting that the association rules could not be effective for some participants.

## Conclusion

This study presented a painting image browser for the interactive exploration based on the categorical information assigned to the images and the numeric feature values calculated from the pixel values of images. The browser employs a multidimensional data visualization technique that highlights the ranges of numeric variables that satisfy the association rules. This paper introduced a case study and an experiment of this browser compared with ImageCube [10]. We concluded that the presented browser performed better evaluations on two factors, overview and comparative browsing, rather than ImageCube.

Extended representations of a large number of numeric variables are one of our future issues. The dataset introduced in this paper was easy to be represented using our browser because it included only 10 numeric variables. Dimension selection algorithms may be effective to visualize only meaningful numeric variables in a limited screen space.

Another interesting problem is the reconsideration of image features. Our current implementation employs a clustering algorithm with some image feature values; thus, it could be more effective if we employ newer clustering mechanisms, such as non-supervised learning using deep neural networks.

After improving the implementation of our technique, we would like to validate our technique with larger datasets. We had a case study and experiments with a mid-size of the dataset that had only 858 images, which were limited to paintings by impressionists. We could not find the sufficiently various association rules with the case study because the number of painting images was not sufficiently large, or categories of the images were unbalanced. Therefore, we intend to test our application again after preparing larger datasets containing more variety of painting images.

## Data Availability

The dataset applied in this paper is not open but authors can provide on demand.

## Abbreviations

ARCP: Association rule control panel
CBIR: Content based image retrieval
CSP: Category selection panel
IFVA: Image feature visualization area
PIDP: Painting image display panel
